# Measurement, Determinants, and Implications of Energy Intake in Athletes

**DOI:** 10.3390/nu11030665

**Published:** 2019-03-19

**Authors:** Bryan Holtzman, Kathryn E. Ackerman

**Affiliations:** 1 Perelman School of Medicine, University of Pennsylvania, Philadelphia, PA 19104, USA; bryan.holtzman@pennmedicine.upenn.edu; 2 Female Athlete Program, Division of Sports Medicine and Endocrinology, Boston Children’s Hospital, Boston, MA 02115, USA; 3 Neuroendocrine Unit, Massachusetts General Hospital, Boston, MA 02114, USA; 4 Harvard Medical School, Boston, MA 02115, USA

**Keywords:** exercise, appetite, ghrelin, leptin, PYY, GLP-1, energy availability, exercise-induced anorexia

## Abstract

Appropriate energy intake is important for the health and performance of athletes. When an athlete’s energy intake is not concordant with energy expenditure, short- and long-term performance can be compromised and negative health effects may arise. The energy intake patterns of athletes are subject to numerous effectors, including exercise response, time, and availability of food. To assess different determinants of energy intake in athletes, we reviewed recent literature regarding the response of appetite-regulating hormones to exercise, appetite perceptions following exercise, chronic exercise-induced adaptations regarding appetite, and social factors regarding energy intake. Additionally, we discussed consequences of aberrant energy intake. The purpose of this review is to clarify understanding about energy intake in athletes and provide insights into methods toward maintaining proper energy intake.

## 1. Introduction

Athletes represent a population with unique energy and nutritional needs, as well as particular barriers to achieving these needs. Sufficient energy availability (EA) is important for optimal health, performance, and injury mitigation [1]. Imbalances in EA arise as a result of insufficient energy intake (EI) to meet the needs of the exerciser.

For quite some time, it has been accepted that acute exercise is not followed by a compensatory increase in hunger and caloric intake [2]. A review from 1999 concluded that hunger and EI do not rise following caloric deficit created by exercise [3], a phenomenon that has been variably termed exercise-induced anorexia [4].

This review discusses various determinants of EI, energy expenditure, and EA in athletes, including some notable endocrinologic responses to exercise, social pressures, and behavioral changes. Furthermore, this report will cover the effects of inadequate EI on health and performance. Sex-differences in the appetite hormone response to exercise in athletes have not been thoroughly studied in a controlled manner; this report will draw on studies of both sexes.

## 2. Measures of Energy Status

Energy balance (EB) is often considered the hallmark of one’s energy status. EB is usually interpreted to be achieved when EI equals energy expenditure—that is, calories in equals calories out. In a research setting, EB is calculated as the difference of EI and total energy expenditure [1]. Total energy expenditure is further broken down into the sum of basal metabolic rate, the thermic effect of food, and the thermic effect of activity [1]. While such a definition is useful in a research setting, recent commentary has argued that this EB equation incorrectly portrays EB as a static system when it is mediated by a number of dynamic relationships in vivo [5]. The response to changes in EB is subject to substantial interindividual variability [6,7,8], and much of the research on EB to this point has focused on single constituents of the system [5].

EA is the preferred marker of energy status for athletes [1]. Female Athlete Triad (Triad) and Relative Energy Deficiency in Sport (RED-S), which describe the negative sequelae experienced by athletes who do not satisfy the energetic needs for their level of physical activity, use EA as the underlying determinant of inadequate vs. replete energy status [9,10,11,12]. EA is calculated as EI minus exercise energy expenditure (EEE), normalized to fat free mass (FFM) per day: $\mathrm{EA}=\frac{\mathrm{EI}\;\left(\mathrm{kcal}\right)-\mathrm{EEE}\;\left(\mathrm{kcal}\right)}{\mathrm{FFM}\;\left(\mathrm{kg}\right)}{\;\mathrm{day}}^{-1}$ [11,13]. EA quantifies the amount of energy available for bodily functions after the energetic cost of exercise has been removed [13] and quantifies the behavior of athletes [14]. EA simplifies the complicated components of EB by only quantifying the amount of energy lost due to exercise and correlating the remaining available energy with physiological functions [15].

EB is intuitive to understand: a value of 0 indicates complete balance, a positive value indicates caloric surplus, and a negative value indicates a caloric deficit. EA, on the other hand, is less intuitive. Traditionally, 30 kcal·kg^−1^ FFM·day^−1^ has been cited as the threshold between normal and abnormal functioning based on disruption of luteinizing hormone (LH) pulsatility in women [14]. Recent data, however, have indicated that menstrual function is disrupted in some women at energy availabilities >30 kcal·kg^−1^ FFM·day^−1^, suggesting that the previously defined threshold of 30 kcal·kg^−1^ FFM·day^−1^ is more fluid or susceptible to interindividual variability, or that such a threshold cannot be defined [16,17]. The EA threshold has not been extensively studied in men, and men lack the surrogate of low EA employed in many studies of women (menstrual dysfunction).

Regardless of the definition of low EA, an energy replete state has been defined as 45 kcal·kg^−1^ FFM·day^−1^ [1,15]. Ranges of EA and their effects on athlete physique are shown in Table 1. While inadequate EA is often the focus of sports physicians and dietitians—particularly those caring for female athletes—some athletes may desire to gain body or muscle mass for the purpose of their sport, and a proper understanding of EA will assist these athletes in achieving their goals.

Accurate assessment of EA in a free-living/field situation, however, is not without its challenges, and there is no standardized method for measuring EA outside the lab. Each component of EA—EI, EEE, and FFM—can be ascertained through several methods at varying costs and accuracies. Most studies have used dietary records of variable lengths, usually 3–7 days, and/or a 24 h dietary recall to assess EI [18]. The major pitfalls of dietary records are an over- or underestimation of EI and an inaccurate representation of long-term EI [19,20,21,22]. EEE has often been calculated using activity log estimates, heart rate monitoring, and/or accelerometry [18]. Wearable devices may over- or underestimate EEE and do not necessarily capture all intentional exercise movements (e.g., if the wearable measures activity by accelerometry and is mounted on the wrist while an athlete cycles) [18,23] and activity logs are considered even less accurate [18]. Estimations of FFM are variable and many tools are available. Some common examples include skin fold measurement, bio-electrical impedance analysis, and dual-energy x-ray absorptiometry (DXA) [24,25]. DXA appears to be the best estimate of body composition in athletes but is still prone to a lack of standardized techniques [26]. Regardless, FFM inaccuracies contribute less noise to EA calculations than the EI and EEE components [18].

## 3. Appetite Regulating Hormone Response to Exercise

### 3.1. Hormone Introduction

Regulation of appetite is subject to many factors that can ultimately result in a person feeling hungry or sated. This section will discuss the impact of acute and chronic exercise on four appetite regulating hormones: leptin, ghrelin, peptide YY (PYY), and glucagon-like peptide-1 (GLP-1). Many other hormones have been implicated in appetite regulation, including cholecystokinin, neuropeptide Y (NPY), insulin, serotonin, glucagon, galanin, enterostatin, adiponectin, oxytocin, and amylin; this review focuses on just a few hormones that have been studied in athletes to demonstrate the complexity of interactions among the endocrine system and exercise [27,28]. The studies discussed in this review and the proposed effects of exercise on each hormone are summarized in Table 2.

Leptin is an adipokine with anorexigenic effects. Leptin levels decrease in both acute and chronic energy deficient settings to enhance food intake [50]. When leptin levels are elevated, satiety is sensed. Leptin levels correlate with absolute fat mass [50] and percent body fat [51] due to a greater presence of leptin-releasing adipocytes. Obese individuals are hypothesized to continue excess energy intake despite these increased leptin levels due to leptin resistance [52]. 

Ghrelin is primarily produced in the fundus of the stomach and exerts orexigenic effects [53]. Upon release from the stomach, ghrelin acts on the hypothalamus to promote gastric acid secretion and feelings of hunger [54]. Interestingly, hypothalamic arcuate cells simultaneously express the leptin and ghrelin receptors [55]. Leptin and ghrelin are classically conceived as oppositional hormones. Those in chronic states of energy deprivation, such as in anorexia nervosa, have higher levels of ghrelin than the energy replete [56]. Because exercise creates a caloric deficit, it would be expected that such exercise would induce a subsequent increase in circulating ghrelin levels. However, the acute response of ghrelin to exercise is equivocal: either its levels are unchanged or, paradoxically, ghrelin levels decrease, as described further below.

PYY is also an anorexigenic hormone acting on the hypothalamus to decrease food intake [28]. Additionally, PYY has functions not related to appetite regulation, including gastrointestinal transit, substrate metabolism, and thermogenesis [35]. Caloric intake induces PYY release from intestinal cells [28]. In patients with anorexia nervosa—a state of chronic energy deficiency—PYY levels have been found to be elevated, unaffected, or decreased [56]; if levels are elevated, such an adaptation may contribute to decreased EI [57]. PYY levels are blunted in obese individuals [58].

GLP-1 is a peptide incretin hormone released by enteroendocrine L cells [59]. The main action of GLP-1 is to maintain glucose homeostasis by promoting insulin release following nutrient consumption [59]. GLP-1 is also hypothesized to play a role in EB through anorectic effects, ultimately localizing to the solitary nucleus of the brainstem [59]. In tests of fasted adolescent female anorexia nervosa patients [mean body mass index (BMI): 14.8 ± 1.4 kg·m^−2^], GLP-1 levels were lower in the anorexia patients than age-matched, normal-weight controls [60]. Meta-analysis revealed that following exercise, GLP-1 levels rose 13.1% compared to non-exercising controls [39].

### 3.2. Exercise Effects on Leptin, Ghrelin, PYY, and GLP-1

In early 2014, a systematic review and meta-analysis was published about the response of appetite regulating hormones to acute exercise [39]. This meta-analysis found that exercise had a median 16.5% suppressive effect on acylated ghrelin, which the authors characterized as a small to moderate decrease [39]. In aggregate, PYY increased a median of 8.9% and GLP-1 increased a median of 13.0%; leptin was not analyzed [39]. The studies included in this meta-analysis involved participants of normal body weight (median BMI: 23.4 kg·m^−2^) and good fitness (median VO_2_max: 56.9 mL·kg^−1^·min^−1^); the majority of the sample (77.6%) were men. A particularly interesting study included in the meta-analysis, as well as more recent studies, are discussed below.

The results of studies of leptin changes in response to exercise seem to follow the predicted response: transient caloric deficiency reduces circulating leptin. A study of trained male cyclists founds that maximal exercise induced a 21.4% decrease in plasma leptin immediately following the completion of the training session and a 21.2% decrease 60 min after the completion of the session [29]. Furthermore, 6 months of cycling training caused a 44.9% decrease in leptin levels (along with decreases in body weight, BMI, and fat mass) [29]. Another study subjected participants to 4 h of 65% VO_2_max treadmill exercise, inducing an average energy expenditure of 3363 ± 254 kcal [30]. At the end of the exercise session, leptin levels had dropped 22.5%, and significant drops in leptin were observed after 2 h of exercise [30]. A study of trained male rowers found that a 2 h training session caused a 23.1% decrease in leptin 30 min after finishing the session [31].

High-intensity interval training (HIIT) has recently received attention as an alternative means to achieving physical activity recommendations. Furthermore, HIIT more accurately represents the type of physical demands often asked of athletes. Inoue et al. recently measured the effects of HIIT sessions on leptin and found that, like endurance training, HIIT induced reductions in leptin [32]. However, a study of professional female basketball and handball players found that leptin levels were not affected by a single moderate aerobic or intensive fitness/speed exercise, measured after 3 months of pre-season preparatory training [36]. The three months of training did reduce plasma leptin, though changes in fat mass over the training block were not recorded (body mass was unchanged) [36]. Loss of body fat could account for leptin decreases. Better controlled studies will reveal whether initiation of training, change in body composition, or training with change in body composition is responsible for leptin drops.

Leptin changes following resistance exercise have also been studied. A study of college-aged fit men (mean VO_2_max: 51 ± 1 mL·kg^−1^·min^−1^) measured leptin following a high volume, heavy resistance training protocol and a non-exercising control session [33]. The training protocol induced an average EEE of 856 ± 114 kcal and the participants were fed meals designed by registered dietitians [33]. Interaction effects found that leptin levels were lower 9–13 h following the exercise session compared to the control session [33]. However, a separate study found no effect of acute resistance training on leptin in sedentary, weight-trained, running-trained, or weight- and running-trained males [37]. This study, though, only subjected the participants to approximately 15 min of resistance training [37] compared to 125 ± 3.4 min in the prior study [33], perhaps suggesting a confounding effect of EEE. A similar study comparing leptin changes achieved through different modalities of resistance training (maximum strength, muscular hypertrophy, strength endurance) found that no resistance training protocol induced changes in leptin immediately following or 30 min after training that were significantly different than the control session [38]. Leptin levels did drop 30 min after exercise compared to baseline starting values, but this change was in concordance with changes in the control trial [38]. Estimated EEE ranged from 231.19 ± 6.8 kcal (maximum strength) to 327.58 ± 12.8 kcal (strength endurance) [38].

Early work examining the ghrelin response to exercise found that ghrelin plasma concentrations were unaffected by exercise [44]. A study of eight healthy male volunteers (mean age: 29.9 ± 1.9 years; mean BMI: 22.5 ± 0.5 kg·m^−2^; mean VO_2_max: 42.2 ± 2.3 mL·kg^−1^·min^−1^) examined the ghrelin response to different exercise intensities [44]. The participants exercised at 50, 70, and 90% of VO_2_max, achieved by treadmill running, on 3 different days, and blood was drawn before, during, and after exercise. Peri- and post-exercise plasma ghrelin concentrations were not different than the pre-exercise concentration for any of the three exercise intensities [44]. A prior study of 8 healthy controls (mean age: 40.4 ± 4.2 years; mean BMI: 23.6 ± 0.5 kg·m^−2^; mean VO_2_max: 41.7 ± 2.6 mL·kg^−1^·min^−1^) similarly found no effect of exercise on ghrelin concentrations when participants were asked to exercise at their lactate threshold [45].

Most studies examining the effects of exercise on ghrelin levels have used exercise intensities ≤75% VO_2_max [39]. Deighton and colleagues compared the gut hormone response to sprint interval exercise and steady state endurance exercise [40]. A sample of 12 healthy males (mean age: 23 ± 3 years; mean BMI: 24.2 ± 2.9 kg·m^−2^; mean VO_2_max: 46.3 ± 10.2 mL·kg^−1^·min^−1^) completed trials (separated by 1 week) of 60 min of continuous cycling at 65% VO_2_max and 6 Wingate tests (with a 4 min recovery period) [40]. Participants completed more work and had higher EEE in the endurance bout than in the sprint bout, however, acylated ghrelin was suppressed more in the sprint trial than in the endurance trial [40]. Conversely, PYY concentrations were elevated after the endurance bout, but not after the sprint bout [40]. Despite these lowered ghrelin levels, compensatory increases in appetite, but not food intake, were observed several hours following the sprint session [40]. The authors followed food intake for 24 h following each session and found that endurance exercise induced a substantial caloric deficit, whereas sprint exercise did not [40].

A study of the ghrelin response to resistance training demonstrated a decrease in ghrelin levels following exercise [41]. In an overnight fasted state, 14 college-aged male athletes completed 3 circuits of 10 exercises of 8–12 repetitions at 60% of one repetition max (1RM). Blood was collected immediately following completion of the circuit and 24 h after exercise. Ghrelin levels dropped immediately post-exercise compared to pre-exercise levels, but 24 h post-exercise ghrelin levels were higher than both pre- and immediate post-exercise values [41]. This stands in contrast to some of the studies discussed, where ghrelin levels were unaffected or diminished 24 h after exercise. This could suggest differing effects of exercise modality, caloric expenditure (not measured in the resistance training study [41]), or food intake in the intervening time between exercise and 24 h measurement.

In comparison to studies of other hormones, there are fewer investigations of the effect of exercise on GLP-1 levels. A study of endurance trained college-aged men (mean BMI: 21.0 ± 1.6 kg·m^−2^; mean VO_2_max: 61.6 ± 6.0 mL·kg^−1^·min^−1^) found that following exercise at 76% VO_2_max for 30 or 45 min rose 29% and 49%, respectively, for up to 60 min post-exercise compared to resting control trials [47]. Subjective appetite and absolute EI were unaffected by exercise; consequently, relative EI in the exercise trials were lower than in the non-exercising trial [47]. In a separate study of endurance-trained women (mean VO_2_max: 55.2 ± 4.3 mL·kg^−1^·min^−1^), GLP-1 rose immediately following exercise completion [48]. The participants were asked to exercise at 60% of VO_2_max (mean duration: 45.7 ± 10.8 min) and 85% of VO_2_max (mean duration: 33.6 ± 5.6 min); exercise intensity did not affect the increase in GLP-1 [48]. GLP-1 levels, however, returned to baseline 60 min post-exercise [48]. Hunger and desire to eat were depressed upon conclusion of the exercise session compared to baseline, though satiety and fullness were lowered and the desire to eat elevated at the 60 min post-exercise timepoint, mirroring the hormone levels [48].

The effect of HIIT on GLP-1 levels has also been studied. Fifteen female collegiate lacrosse players completed trials of 8 × 6 s sprints on a cycle ergometer, separated by 30 s of rest [49]. GLP-1 levels were unaffected by the exercise session at measurements at completion of the trial and 30 min after the trial [49]. EI at an ad libitum buffet meal following the exercise session was significantly lower in the exercise trial compared to a control trial [49]. The effects of HIIT and resistance training on GLP-1 levels remains a ripe area for study.

Recent studies have examined the effects of an acute bout of exercise on appetite regulating hormones and appetite for up to 24 h after exercise, as well as the effects of repeated exercise. Douglas and colleagues had participants exercise for 60 min at 70% VO_2_max on a treadmill for two consecutive days and assessed appetite, food intake, and appetite regulating hormones [35]. Fasting leptin was unaffected the morning after the first day of exercise and leptin levels compared to control trials (days without exercise separated from experimental trials by at least a week) were unchanged after the second exercise session [35]. Ghrelin was unaffected by exercise, however, hunger ratings and relative EI were suppressed [35]. PYY was transiently elevated following the second exercise session, and the authors noted that the expected physiological change would be a downregulation of PYY to allow for compensatory EI; they suggested that the non-appetite functions of PYY may predominate in the response to exercise [35]. In all, the authors concluded that isolated high volume exercise does not induce compensatory appetite regulation changes [35]. King et al. examined the appetite effects of exercise up to 24 h after an exercise session [34]. Young, healthy male volunteers completed 90 min of treadmill running at 70% of their VO_2_max. Plasma leptin area under the curve (AUC) was reduced for the entire day following exercise compared to a control trial (separated by at least a week), an expected response to caloric deficit [34]. On the day after exercise, plasma acylated ghrelin levels were the same as they were in control trials, despite the caloric deficit induced by exercise (absolute EI was the same between trials) [34]. Post-prandial PYY concentrations, however, were unaffected by the previous day’s exercise and were similar to control [34]. Additionally, fasting appetite perceptions on the day after exercise were not affected by exercise, and interestingly, plasma acylated ghrelin levels were lower after lunch the day after exercise when compared to controls [34]. These results suggest an uncoupling of exercise and hunger perceptions as well as a mismatch between EEE and ghrelin response.

Broom et al. investigated whether exercise intensity or duration played a larger role in determining the ghrelin response to exercise [42]. One group completed two separate bouts of treadmill running—one at 50% VO_2_max (MOD) and one at 75% VO_2_max (VIG)—at isocaloric EEE, as well as a non-exercise control day, all separated by at least a week [42]. The second group completed treadmill runs of 45 min (EX45) and 90 min (EX90) at 70% VO_2_max and also completed a control day; all sessions were separated by at least a week [42]. Change in acylated ghrelin concentration was lower in VIG than MOD; caloric expenditure for the trials was equal [42]. In examining the effects of duration, EX45 and EX90 both had lower changes in acylated ghrelin concentrations than control, however, EX45 and EX90 had similarly decreased acylated ghrelin concentrations despite EX90 resulting in over twice the energy expenditure of EX45 [42]. Similarly, hunger perceptions were lower in both EX45 and EX90 compared to control and were not different between EX45 and EX90 [42]. The authors concluded that vigorous exercise suppresses acylated ghrelin concentration compared to moderate exercise of equal energy expenditure, and acylated ghrelin and hunger were suppressed longer with increased exercise duration [42]. A separate study examined the effects of modest caloric deficit due to exercise on appetite, ghrelin, and PYY [46]. Participants exercised for 30 min at 64.5% of VO_2_max—an intensity and duration in line with the American College of Sports Medicine (ACSM) guidelines (150 min of moderate to vigorous exercise per week) [61,62] and the new Physical Activity Guidelines for Americans released by the US Department of Health and Human Services (150 min of moderate exercise or 75 min of vigorous exercise per week) [63]. This study found that an exercise-induced caloric deficit caused elevated PYY_3-36_ (the most common circulating form of PYY) levels, but did not change plasma acylated ghrelin, and food intake was unaffected [46]. This indicates that, at least transiently, exercise could induce weight loss because of changes in hunger cues.

Recently, Alajmi and colleagues compared the effects of exercise and food deprivation on appetite regulating hormones, appetite perceptions, and EI between men and women [43]. Like men, women had suppressed acylated ghrelin concentrations following exercise and did not change EI; however, women had overall higher acylated ghrelin levels than men [43]. Appetite response and PYY response to exercise were no different between men and women [43].

## 4. Appetite and Food Intake in Response to Chronic Exercise

A number of the studies discussed above measured appetite and EI, and found that both were suppressed following exercise. While an acute bout of exercise may induce appetite suppression, a common question is whether the decreased EI is transient, compensated for days or weeks after exercise, or if the effect is chronic. Research on this topic in athletes is sparse [64].

One study measured EI compensation of moderate and high intensity exercise interventions lasting 14 days [65]. On average, participants increased their caloric intake and compensated for 30% of their elevated EEE over those 14 days [65]. When the researchers removed individuals who were less compliant with the study’s diet, compensation increased to 56%, however, this subset analysis nearly halved the sample size [65]. Another group compared energy compensation of 4 weeks of sprint-interval exercise to HIIT [66]. At the conclusion of the intervention, individual compensation levels were highly variable, ranging from −2081% to +805%, however, 66.7% of the participants did not fully compensate while 33.3% overcompensated [66]. Interestingly—and, perhaps, concerningly—those who undercompensated had greater increases in VO_2_max following the training block than those who overcompensated [66]. Participants in this study were moderately active, though slightly overweight (mean BMI: 27.7 ±4.2 kg·m^−2^) [66].

A study compared the adipokine profiles of sedentary, weight-trained, running-trained, and weight- and running-trained men [37]. Trained men had lower baseline levels of leptin than the sedentary men, an effect that may be due to reduced adiposity [37]. This remains an area of research warranting further investigation.

## 5. Challenges of Achieving Proper Energy Intake for Athletes

Athletes face a number of barriers to achieving proper EA and nutritional intake, and improper diet can lead to a spectrum of effects, ranging from insufficient EA and inappropriate weight loss to unintended weight gain [67]. Athletes who are better informed about proper nutrition tend to make better dietary decisions than their uninformed counterparts [68], though data regarding this issue are mostly derived from unvalidated surveys [69]. Nevertheless, dietary strategies depicted in the popular media are filled with intrigue and innuendo, and access and awareness of data-driven nutritional practices remains an issue among athletes. College athletes counseled by a registered sports dietitian have better nutritional knowledge than those who are not [70,71], though this knowledge does not always translate to better dietary practices [68,72]. Access to a dietitian can be difficult—only 99 NCAA Division I schools employ a sports dietitian, and at most schools, one dietitian serves the entire varsity athlete population [73]. At the high school level, athletes usually need to privately seek out a dietitian at a cost that may be prohibitive for longitudinal oversight.

A study employing focus groups identified time and financial constraints, lifestyle, physique, and nutrition knowledge as the chief barriers to athletes achieving an appropriate diet [74]. Furthermore, an athlete’s programming is often designed by a coach and the athlete may be unaware of the week’s training or changes to the plan—even if the athlete’s meals are guided by a nutritionist, this lack of knowledge may prevent the implementation of a proper nutrition plan. Athletes responsible for supporting themselves financially—such as college athletes—may be unable to afford healthy foods [75]. Recent changes to NCAA rules allowing schools to provide more food to their athletes may improve food availability pertaining to financial constraints.

High school, college, and professional athletes often have many obligations taking time away from nutritional fulfillment. Because of these pressures, it has been reported that athletes prefer convenient or prepared food [76,77,78]. Convenience becomes particularly important when traveling, as foods available at competition arenas often do not fit the needs or preferences of athletes [79], yet those foods are readily available. Furthermore, elite athletes have been shown to prefer foods familiar to them, a complicating factor when traveling internationally [80]. For example, Usain Bolt famously claimed to subsist nearly entirely off of McDonalds at the 2008 Beijing Olympic Games due to his unfamiliarity with Chinese food [81].

While physique is important in nearly all sports, athletes in sports emphasizing leanness face extra challenges in maintaining proper nutrition. “Leanness” sports have been defined as endurance sports (e.g., distance running), sports with an aesthetic component (e.g., dance), weight class sports (e.g., judo), and antigravitation sports (e.g., gymnastics) [82]. Coaches who lack nutritional knowledge may instruct athletes to lose weight, which can trigger disordered eating and eating disorders [83,84]. Furthermore, leanness athletes of all competitive levels (recreational to elite) have been shown to have higher disordered eating tendencies than their non-leanness peers [84]. Involvement of a Board Certified Specialist in Sports Dietetics (CSSD) in treating an athlete with an eating disorder is an important component to recovery [1,9,12,85]. Conversely, in some sports (e.g., American football, Sumo wrestling, downhill sports), athletes may benefit from higher weight or body fat, and this can cause other adverse health effects, such as cardiovascular disease risk factors, obesity, and musculoskeletal injuries [86]. Nutritional behaviors in this population remains an emerging area of research.

## 6. Aberrant Energy Intake: Low Energy Availability

A full review of the effects of low EA is beyond the scope of this report, but an overview of consequences of low EA in athletes warrants discussion. Female athlete triad is the interrelationship of EA, menstrual function, and bone health [9,11]. The severity of disease is assessed through each independent component, though deficits in one component can be detrimental to others [9]. RED-S includes the components of Triad but is centered on low EA, expanding the effects of low EA to other physiological systems: endocrine, immunological, cardiovascular, hematological, psychological, gastrointestinal, growth and development, and metabolic [10,12]. The Triad model includes these proposed effects as secondary sequelae, but research has largely focused on the three main Triad components [11].

One of the most important innovations of the RED-S model was the inclusion of men as a population at risk of low EA [12]. Men suffering from low EA remains an understudied population and the pervasiveness of low EA in general and in specific sports is largely unknown [87]. Furthermore, the health effects of low EA in men are less established than in women [87]. The RED-S model proposes shared consequences of low EA [12], though low EA could affect different systems in men than in women [88] and there is not yet a proposed threshold of low EA for men [87]. Men cannot suffer from menstrual dysfunction, however, work pioneered by Hackney has identified a corollary in men termed the “exercise-hypogonadal male condition” [89]. Similar effects of low EA in Triad have been identified and reviewed in males [90] and recent research has identified that bone stress injuries occur more often in men who have elevated Triad risk assessment scores (modified for men) [91]. Proposals of a “male athlete triad” are in their infancy [92].

Regardless of terminology, low EA underpins many negative health effects and, as depicted by the RED-S model, harms an athlete’s performance [12]. Mathematically, low EA results from insufficient EI to meet exercising needs. The endocrine effects of low EA have been reviewed extensively by Elliot-Sale and colleagues [28]. As relevant to the discussion of EI, chronic low EA causes decreased leptin, increased ghrelin, increased PYY, decreased insulin, and decreased amylin [28]. It is worth noting, however, that the literature reviewed by this study included surrogates of low EA, such as anorexia nervosa and menstrual dysfunction. Therefore, elucidating the exact influence of chronic low EA on the hormones is difficult, though illustrative of the interconnected nature of the pathologies. As reviewed in this report, leptin and PYY levels respond to chronic low EA similarly to acute exercise-induced caloric deficit, yet ghrelin levels, which decreased immediately following exercise, are, as expected, elevated in chronic low EA [28]. One could hypothesize that ghrelin levels following exercise contribute to the development of low EA.

## 7. Conclusions

Energy intake in athletes is subject to a vast number of factors, ranging from hormonal to behavioral and environmental factors. The ultimate determination of meal frequency and amount is complex, and no one factor can perfectly predict or change EI. The transient suppression of ghrelin and appetite following exercise is particularly interesting, indicating that EI is not simply governed by caloric deficit. Furthermore, protein intake early in the post-exercise window and carbohydrate intake throughout the post-exercise window are important for recovery from exercise and muscle anabolism [1], yet the appetite and ghrelin suppressive effects of exercise may prevent athletes from optimizing their nutritional response to exercise.

When considering the determinants of EI, it is important to remember that feeding habits are developed over years [3]. When EEE habits change, such as undertaking a higher training load, EI compensation may be delayed. Consultation with a sports dietitian for guidance on proper fueling for sport can optimize nutritional intake and prevent under- or overeating. Due to the challenges of measuring EA in a free-living environment, athletes and their entourages should be aware of the negative consequences of low EA and use the presentation of those changes as a symptom of low EA.

Research regarding EI is ongoing and will remain a fertile field of academic interest. Future studies should examine overweight yet fit athletes, the physiological changes of males exposed to chronic low EA, and intervention strategies to maintain optimal EI and EA in athletes who do not have access to a sports dietitian.

## Figures and Tables

**Table 1:** Loucks’ proposed energy availability ranges for different athletic functions. Adapted from Loucks, 2013 [15].

EA Range	Effect on Body Mass/Composition
>45 kcal/kg FFM/day	Gain of body mass, muscle hypertrophy, carbohydrate loading
(>188 kJ/kg FFM/day)	
~45 kcal/kg FFM/day	Maintenance of body size and mass; focus on skill development
(188 kJ/kg FFM/day)	
30–45 kcal/kg FFM/day	Loss of body mass or fat
(125–188 kJ/kg FFM/day)	

**Table 2:** Summary of discussed appetite regulating hormones’ response to exercise.

	Increased	Decreased	Unchanged
**Leptin**		[29,30,31,32,33,34]	[35,36,37,38]
**Ghrelin**		[39,40,41,42,43]	[34,44,45,46]
**PYY**	[39,40,46]		[34,40]
**GLP-1**	[39,47,48]		[49]
